# Sourcing Herod the Great's calcite-alabaster bathtubs by a multi-analytic approach

**DOI:** 10.1038/s41598-022-11651-5

**Published:** 2022-05-07

**Authors:** Ayala Amir, Amos Frumkin, Boaz Zissu, Aren M. Maeir, Gil Goobes, Amnon Albeck

**Affiliations:** 1 Martin (Szusz) Department of Land of Israel Studies and Archaeology, Bar-Ilan University, 5290002 Ramat Gan, Israel; 2 Institute of Earth Sciences, The Hebrew University of Jerusalem, 91904 Jerusalem, Israel; 3 Department of Chemistry, Bar-Ilan University, 5290002 Ramat Gan, Israel; 4 Present Address: The Sonia and Marco Nadler Institute of Archaeology, Tel Aviv University, 69978 Tel Aviv-Yafo, Israel

**Keywords:** Chemistry, Analytical chemistry

## Abstract

Herod “the Great”, king of Judea in the second half of the first century BC, was known for his building projects, wealth, and political power. Two of his personal calcite-alabaster bathtubs, found in the Kypros fortress and the palace of Herodium, are among the very limited archaeological evidence of his private life. It seemed plausible that they were imported from Egypt, the main source of calcite-alabaster in ancient periods. Yet, the recent identification of a calcite quarry in the Te’omim cave, Israel, challenges this hypothesis. Here, we developed an approach for identification of the source of calcite-alabaster, by combination of four analytical methods: ICP, FTIR, ssNMR and isotope ratio. These methods were then applied to Herod’s bathtubs demonstrating that they were indeed quarried in Israel rather than in Egypt.

## Introduction

Herod, known as “Herod the Great,” was born ca. 73 BCE, during the reign of the Hasmonean queen, Alexandra (Salome). He was appointed king of Judea at Marcus Antonius's behest and by the Roman Senate in 40 BCE^1^, but managed to establish his reign over Judea only three years later^2^. His authority was approved by Octavian (later Augustus) in 31 BCE. As a Roman client king, Herod was dutiful to the Roman Caesar Augustus, and eager to present himself as a champion of the traditional values of Hellenistic and Roman monarchy, whose influence can clearly be detected in many of his building projects^1^. Furthermore, he adopted Roman cultural norms, such as his introduction of Roman bathing culture into his realm^3^. The western fascination with Herod the Great derives both from his notoriety in Christian tradition for ‘the Massacre of Innocents’ as a deliberate attempt to kill the infant Jesus (Matthew 2: 16)^4^, and from his remarkable success in establishing a quite long monarchy, navigating the different powers and interests affecting his realm. Herod’s “greatness,” reflected in his building projects, his wealth and political power, was known worldwide. He was a prodigious builder, constructing fortresses, palaces and entire cities in Judea, such as Masada, Jericho, Kypros, Paneas (Caesarea-Philippi), Caesarea (Maritima), Jerusalem and Herodium, but also endowing architectural and cultural projects outside his realm. Few individuals were so influential in changing the material culture of their time as he was; in fact, he introduced new trends in almost every aspect of life in Judea as documented by archaeology^5^. The exciting recent discovery of Herod's tomb at Herodium, southeast of Jerusalem, aroused new interest in his life and death. While much is known about Herod’s public activities, little archaeological evidence of his personal life and belongings is available.

**Figure 1:**
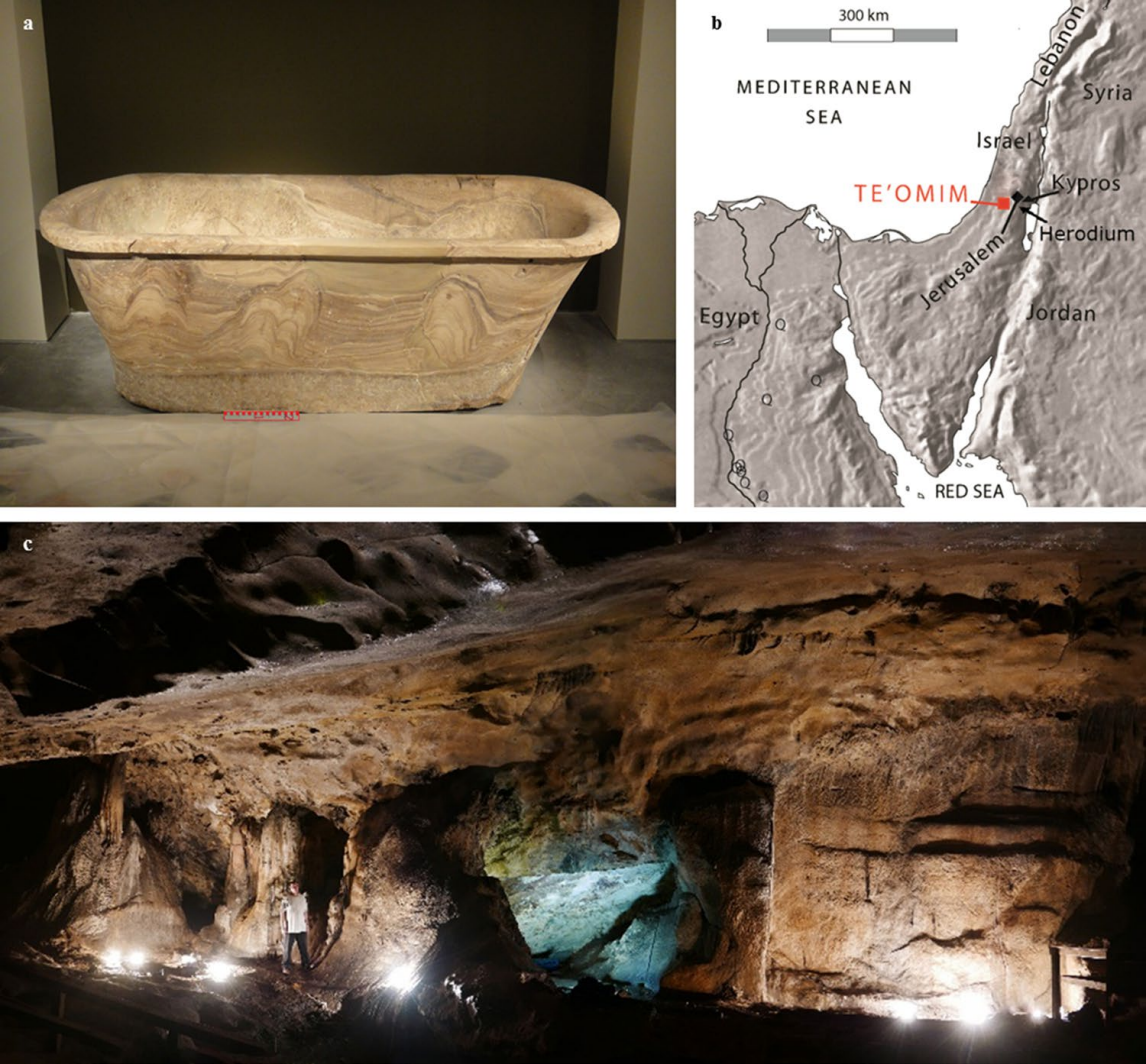
Calcite-alabaster in Israel. (**a**) Herod’s calcite-alabaster bathtub found in Kypros fortress. Modified after ref. 15. (**b**) Location map. The quarry and royal bathtubs studied here are at Te’omim cave, kypros and Herodium. Q indicates calcite-alabaster quarries in Egypt. Modified after ref. 15. (**c**) Te’omim cave. The quarry is located in the right part of the photograph. Photographed by Ayala Amir, Martin (Szusz) Department of Land of Israel Studies and Archaeology, Bar-Ilan University.

Two exceptions to this are Herod’s personal calcite-alabaster bathtubs, one found over 4 decades ago in the fortress at Kypros (Fig. 1a)^6^ and one recently discovered in Herod’s private suite in the palace at Herodium. Both bathtubs were revealed in Herodian contexts which were clearly dated to Herod’s time^7–10^. Where were these bathtubs quarried? Since the Middle Bronze Age, Egypt played a crucial role in the arrival of calcite-alabaster artifacts in Israel, and the development of the local calcite- and gypsum- alabaster industry^11–14^. Therefore, it seemed safe to assume that these bathtubs were imported from Egypt, though transport of these stone tubs would be a challenging endeavor, considering their weight, estimated at 1.5 metric tons each^6^. Their origin has been now revisited with the recent identification of a calcite-alabaster quarry in the Te’omim cave, located on the western slopes of the Jerusalem hills (near modern-day Beth Shemesh, Israel), which was within the boundaries of Herod’s kingdom (Fig. 1b,c)^15^. In this cave, signs of quarrying-scars and cessation of quarrying are visible on the quarry’s walls and floor. Evidence includes ‘negatives’ left after the removal of calcite-alabaster blocks, blocks of calcite-alabaster that were left in place, due to fissures or defects in the bedrock, and cleaving channels—shallow channels left in the calcite-alabaster after a block was separated. U-Th dating results, as well as associated archaeological finds, indicate that major quarrying activities took place in the Te’omim Cave prior to 1500 BCE, during the Middle Bronze Age. Later use of the quarry is indicated by an abundance of Late Roman remains that were found on the quarried surfaces in the Cave^15,16^. These results are noteworthy, as it was previously assumed that all calcite-alabaster found in the Levant originated from Egypt, while poorer quality vessels made of gypsum were local products^11^. Based on these data, it is possible that Herod’s bathtubs were carved and made locally, in Judea, rather than in Egypt. In this study we address this exciting new possibility, and show through chemical analysis that they were indeed local in origin.

The present research joins previous archaeometric and geologic studies of calcite-alabaster originated in the Mediterranean Basin, previously reviewed by Koralay et al.^17^ and Scardozzi et al.^18^. The former studies involved minero-petrographic and trace-element analyses, as well as δ^13^C, δ^18^O, δ^87^Sr isotopic measurements for provenance determination. Solid-state ^13^C-NMR was also argued to be successful for this analysis, by comparison of the intensities and line-widths of ^13^C-NMR profiles^19^.

Since Egypt was the main source of calcite-alabaster artifacts in Israel (see above), the focus of the present study, we compare Israeli samples mainly to those of Egyptian source, rather than to other Mediterranean sources for which there is no documentation of trade in calcite-alabaster with Israel.

Petrographic analysis of regional^20^ and Te’omim Cave^21^ speleothems (calcareous chemical cave deposits) shows wide variability in texture, depending on depositional environment within the cave and general climatic controls. Our visual inspection of the Herodian bathtub shows similar textural variations, from massive flowstone deposits to laminar stalagmitic calcite. While these variations seem to be similar in the bathtub and the regional speleothems, they do not allow accurate provenance determination. Solid-state ^13^C-NMR measurements of samples of the current research did not show significant differences between the Israeli and the Egyptian samples. Consequently, this method could not be used to identify the bathtubs source.

Therefore, in the present study we developed a new approach for the discrimination between different calcite-alabaster sources in order to identify the provenance of Herod’s royal bathtubs. Furthermore, it could be applied to the study of other archaeological calcite-alabaster artifacts. This approach provides information concerning both the composition and crystalline structure of calcite-alabaster and is based on four analytic methods, most of which have not been previously used for provenance determination. The combination of these methods provides much higher confidence in the interpretation of the provided data.

## Results

### Development of methodology

In order to identify the origin of Herod’s bathtubs, we applied a variety of analytical tools that enabled differentiation between calcite-alabaster of Egyptian versus southern Levantine origin. These methods were then applied to determine the origin of Herod’s calcite-alabaster bathtubs. The methods used in this research include: inductively coupled plasma (ICP) analysis, routine infra-red (IR) spectroscopy, ^1^H- and ^31^P- solid state NMR (ssNMR) experiments and C and O stable isotope ratio analysis (see “Materials and methods”).

Analytical data were first collected from samples of two well-defined sources, from Egypt and modern-day Israel. The Egyptian sources included both ancient and modern calcite-alabaster samples. The ancient samples were courtesy of the Kunsthistorisches Museum in Vienna. These ancient vessel remains were collected by the Austrian archaeological expedition to Giza in the nineteenth century. The modern Egyptian artifact (made of geological-sourced calcite-alabaster) was bought in a market in Cairo, Egypt in 2013. The calcite-alabaster from Israel included raw material from the Te’omim cave quarry, chips (mining debitage) found in the cave near the quarry, and chips and a stone block (raw material carved to a cube, but not yet used to make a vessel) from Umm el-‘Umdan—an archaeological site near the Te’omim cave. Additional samples were collected from a speleothem in Natuf cave (located in Wadi en-Natuf in the western Samaria area) (see Supplementary Table S1 online).

### Analysis

**Figure 2:**
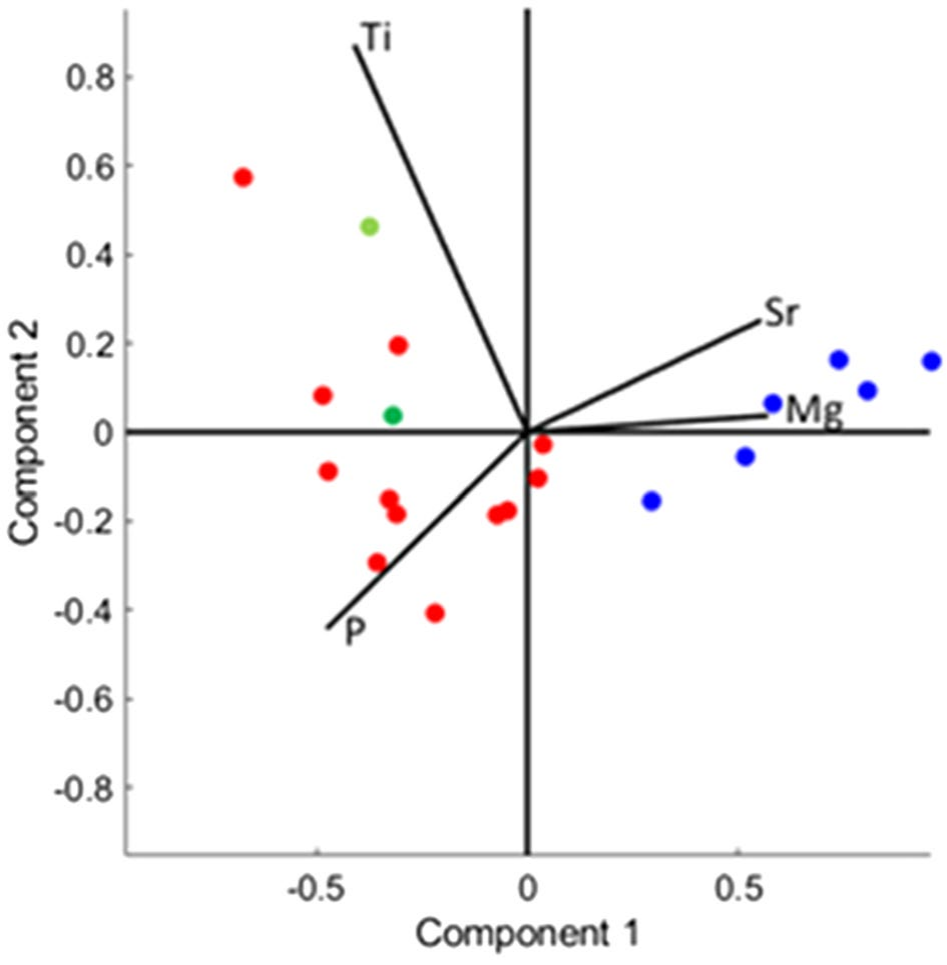
PCA (principal components analysis) presentation of the ICP results. Clustering of the element distribution of the Israeli (red) and Egyptian (blue) samples. The bathtubs data points are in green.

The calcite-alabaster samples from sources in Israel and Egypt were analyzed to determine their composition and their crystalline structure. ICP-AES analysis showed that the distribution of concentrations of the trace elements magnesium, strontium, phosphorus and titanium differed between these samples. Egyptian samples contained significantly higher concentrations of magnesium and strontium compared to the samples from Israel, while their phosphorus and titanium concentrations were lower (Table 1). PCA (principal components analysis) of these data presents clear clustering of the Israeli and Egyptian samples (Fig. 2). The magnesium and strontium concentrations of the Egyptian samples fall within the range of concentrations reported for samples from various quarries in Egypt^22–24^.

**Table 1:** ICP-AES results (in ppm).

Source		Sample #	Mg	Sr	P	Ti
**Israel**	**Te’omim cave chip**	**I-1**	**4016**	**46.6**	–*	**4.446**
	**Te’omim cave quarry**	**I-2**	**6471**	**31.4**	**104**	**0.538**
		**I-3**	**6612**	**33.4**	**70.2**	**0.529**
		**I-4**	**6201**	**30.6**	**92.8**	**0.461**
		**I-5**	**5470**	**28.8**	**42.8**	**0.591**
		**I-6**	**3767**	**40.6**	**161**	**0.708**
		**I-7**	**3361**	**36.2**	**130**	**1.022**
		**I-8**	**3024**	**50.6**	**141**	**2.202**
	**Umm el- ‘Umdan chip**	**I-9**	**4817**	**45.8**	**149**	**1.056**
		**I-10**	**6880**	**34.2**	**124**	**2.494**
	**Umm el- ‘Umdan block**	**I-11**	**6757**	**43.4**	**176**	**0.312**
	**Natuf cave**	**I-12**	**2565**	**42.6**	–*	**1.557**
**Average ± standard deviation**			**4995 ± 1530**	**38.68 ± 6.87**	**119.08 ± 39.55**	**1.326 ± 1.156**
*Egypt*	*Giza*	*E-1*	*12,407*	*836*	*49.3*	*0.597*
		*E-2*	*10,680*	*570*	*38.6*	*0.328*
		*E-3*	*13,427*	*1001*	*30.8*	*0.188*
		*E-4*	*10,002*	*495*	–*	*0*
		*E-5*	*7878*	*392*	–*	*0*
	*Modern raw material*	*E-8*	*13,687*	*653*	*25.9*	*0.192*
*Average* ± *standard deviation*			*11,347* ± *2050*	*657.8* ± *205.9*	*36.2* ± *8.8*	*0.217* ± *0.190*

Israeli samples in bold, Egyptian samples in italics.

*Data not collected.

**Figure 3:**
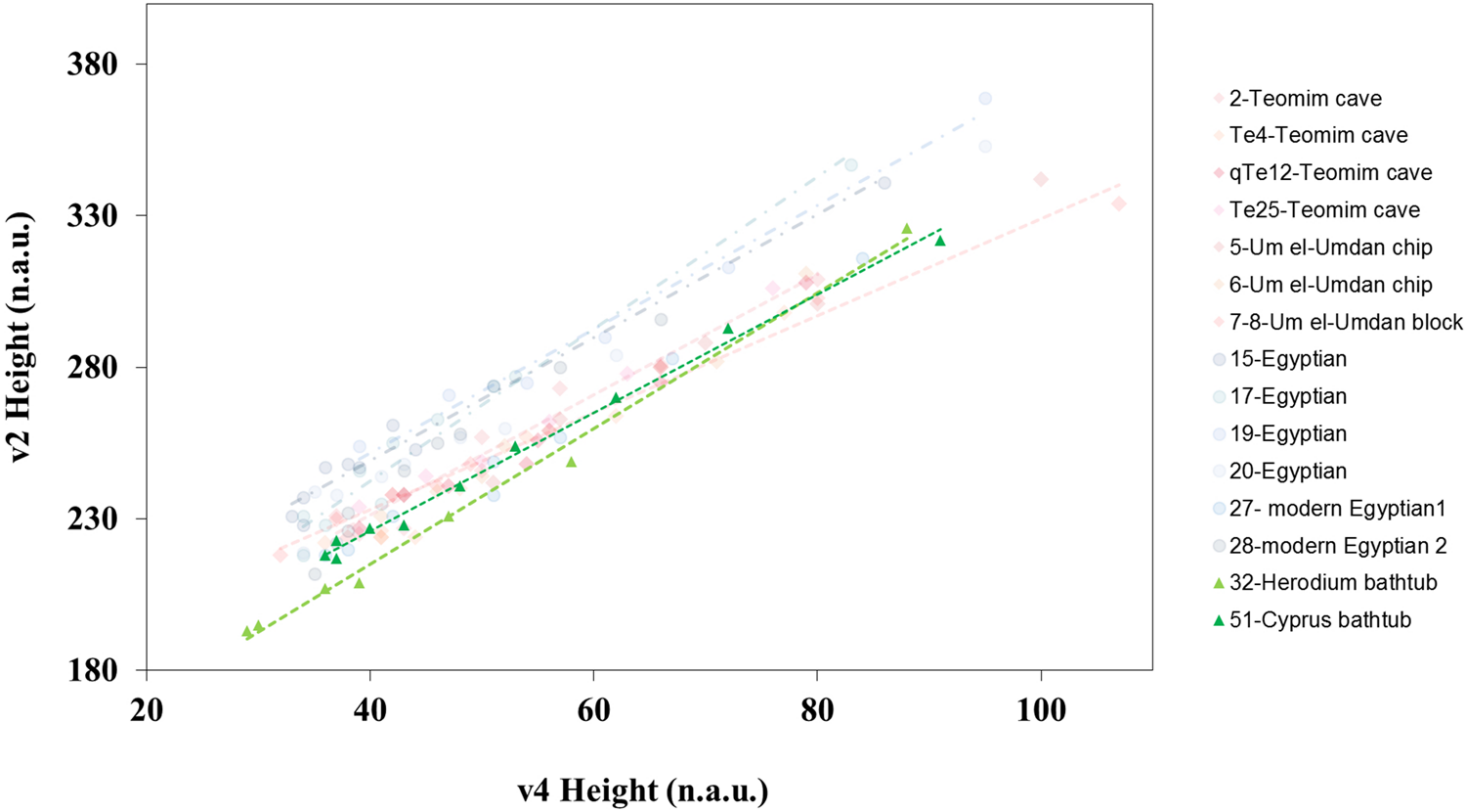
Routine FT-IR grinding curve results of the two bathtubs (triangles) over the background of the Israeli (diamond) and Egyptian (circle) curves.

Routine FTIR analysis^25^ results obtained from repeated grinding between measurements, enabled detection of slight differences in the structure of calcite from various sources (Egypt and Israel). These routine IR tests revealed small changes in the calcite peaks, manifested by different "grinding curves," in terms of location and slope in the graph. The graphs show a clear division between the "Israeli" and "Egyptian" curves; the former are displaced, for the most part, to the right of the latter (with higher v4 values relative to their v2 values), and are characterized by slopes which are more moderate than the Egyptian ones (0.14 ± 1.91 for the former vs. 0.25 ± 2.22 for the latter, Fig. 3). These results indicate a higher degree of atomic order of the calcite-alabaster from Israel in comparison to the Egyptian material.

**Figure 4:**
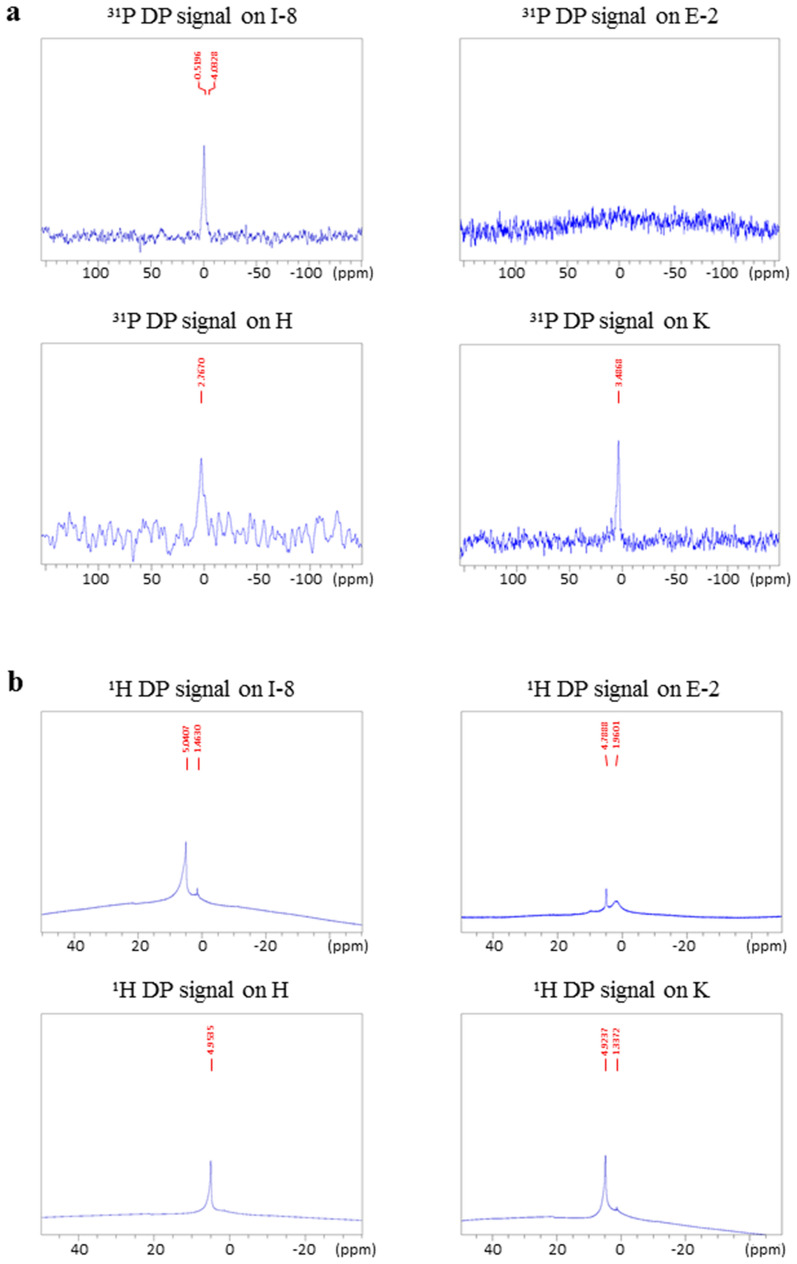
Solid state NMR results: I-8 representative sample from Te’omim cave, Israel; E-2 representative sample from Egypt; H from Herodium bathtub; K from Kypros bathtub. (**a**) ^31^P NMR data. (**b**) ^1^H NMR data.

Solid-state phosphorus (^31^P) and proton (^1^H) NMR analyses were next performed to provide quantitative and structural information regarding the calcite-alabaster. The direct polarization (DP) phosphorus NMR measurements showed clear phosphate peaks in all the samples from Israel, while these peaks were absent from the Egyptian calcite-alabaster (Fig. 4a and Supplementary Fig. S1 online). Thus, the Israeli samples contain phosphorus, whereas in the Egyptian samples, phosphorus content, if present, is below detection level. In addition, the results of the proton NMR measurements show that calcite-alabaster samples from Israeli origin contain relatively more water, both in the bulk of the material (indicated by the broad component of the water peak), as well as on its surface (the narrow component of the water peak) (Fig. 4b and Supplementary Fig. S1 online). This is manifested quantitatively by the ratio of the left peak(s) intensity to that of the right peak (calculated on deconvoluted spectra). Thus, in the samples from Israel this ratio is 7.31 ± 2.25, whereas in the Egyptian samples it is 0.32 ± 0.22. This information is consistent with the results of the ICP and IR analyses.

**Figure 5:**
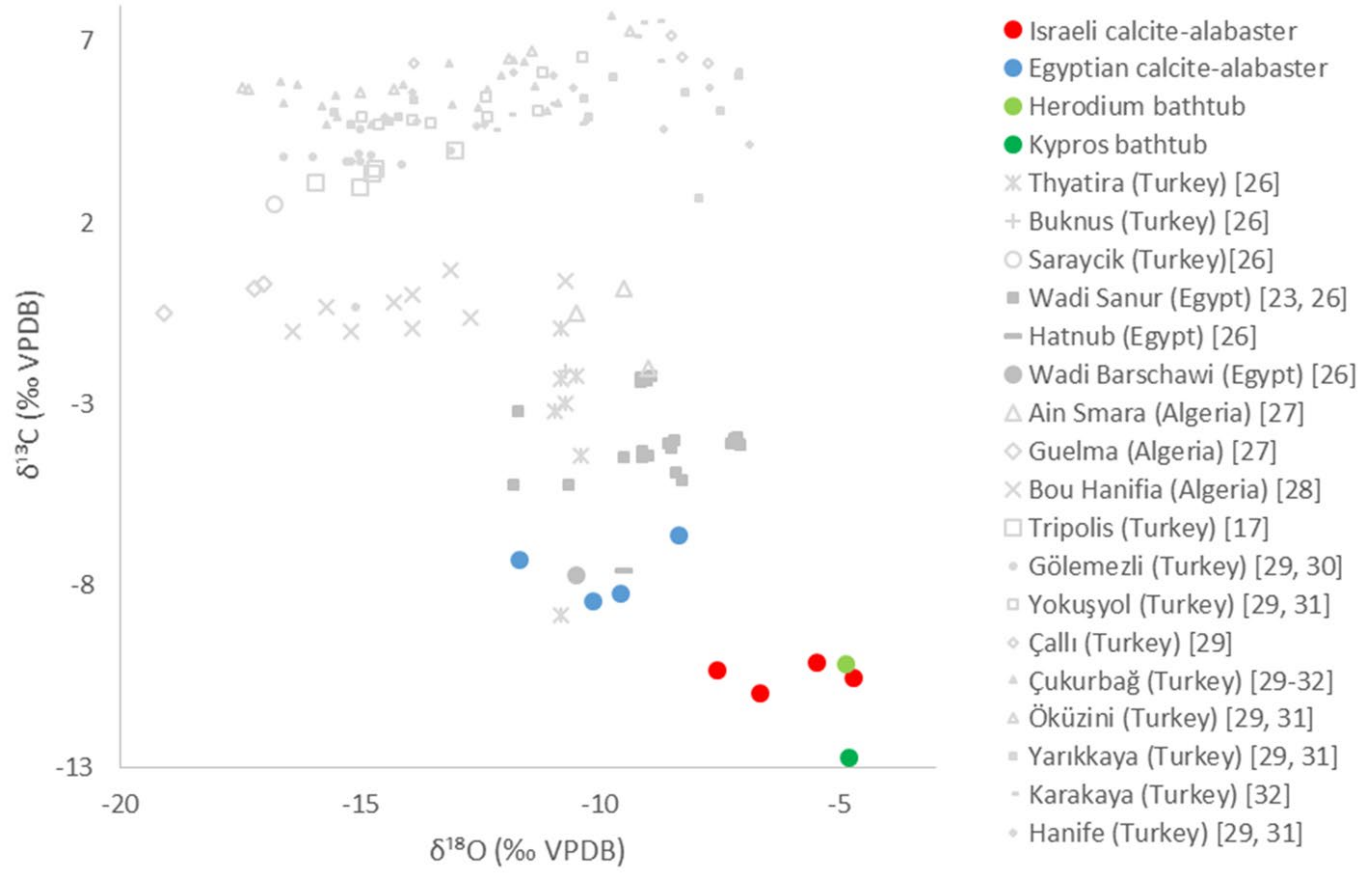
The δ^13^C and δ^18^O stable isotopes diagram. Distribution of the Israeli (red) and Egyptian (blue) samples of the present study, and literature data from the Mediterranean Basin (gray). Herod’s bathtubs data are in green. The numbers in parentheses refer to the reference from which the data was taken.

Finally, we measured carbon (δ^13^C) and oxygen (δ^18^O) isotopic ratios of various samples. This analysis also showed a distinction between the samples from Egypt and Israel (Table 2 and Fig. 5). Furthermore, the Israeli data is distinct from parallel data of calcite-alabaster quarries around the Mediterranean Basin^17,18,23,26–32^ (Fig. 5).

**Table 2:** Carbon (δ^13^C) and oxygen (δ^18^O) stable isotopic ratios.

Source	Sample #	δ^13^C‰	δ^18^O‰
**Israel**			
**Te’omim cave**	**I-5**	**− 10.35**	**− 7.54**
**Umm el- ‘Umdan chip**	**I-10**	**− 10.14**	**− 5.48**
**Umm el- ‘Umdan block**	**I-11**	**− 10.98**	**− 6.67**
**Natuf cave**	**I-12**	**− 10.54**	**− 4.71**
**Israeli isotope ratios (the range of negative values)**		**10.14–10.98**	**4.71–7.74**
***Egypt***			
*Giza (ancient)*	*E-7*	*− 8.45*	*− 10.13*
	*E-2*	*− 6.62*	*− 8.35*
	*E-5*	*− 7.30*	*− 11.67*
*Raw material (modern)*	*E-8*	*− 8.20*	*− 9.56*
*Egyptian Isotope ratios (the range of negative values)*		6.62–8.45	8.35–11.67

Israeli samples in bold, Egyptian samples in italics.

Comparison of data previously collected in Israel^33–36^ and Egypt^23,26^ demonstrates that the corresponding O isotopic ratios partially overlap, whereas the C isotopic ratios of the Egyptian samples fall in a distinct sub-range within the Israeli data. Indeed, the data collected in our experiments correspond well with these published results. Moreover, our results from the Israeli samples fall within the wide literature range for Israeli samples, and are completely distinct from the Egyptian range. We note that our results for both C and O ratios (Table 2) correspond perfectly to the section of the glacial period, stage 6 (ca 130,000–170,000 years ago), measured along a stalagmite spanning the last 170,000 years in a cave in the Judean hills near Jerusalem^34^.

Thus, all four analytical methods applied in this study gave consistent results, clearly distinguishing the Israeli from the Egyptian calcite-alabaster.

### Herod’s bathtubs

The four analytical methods (ICP, routine IR, ss NMR, and isotope ratio) were then applied to samples from Herod’s bathtubs. The vast majority of the analytic data from both bathtubs, obtained through these four analytical methods, clearly fall within the range of the Israeli samples and out of the range of the Egyptian ones (Table 3 and Figs. 2, 3, 4 and 5).

**Table 3:**
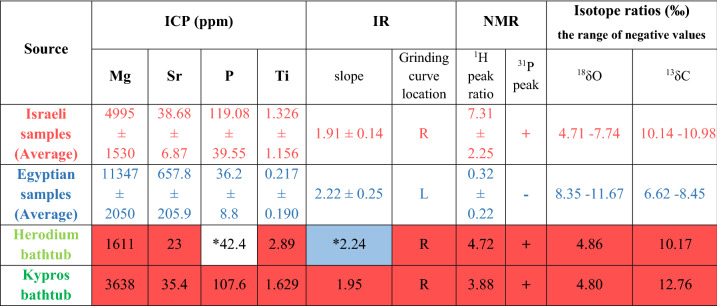
Summary of results.

Israeli samples in red, Egyptian samples in blue. The color coding of the cells in the table showing bathtub samples indicates their similarity to the corresponding values of the "known" Israeli and Egyptian samples. *see text.

Overall, the results unequivocally indicate that the bathtubs were quarried in Israel and not in Egypt.

Two of the data points, measured for the bathtub from Herodium, require clarification. The phosphorus content of the sample from this bathtub is low, within the value range of the Egyptian samples, though it also overlaps the lower level of the Israeli samples (the concentration ranges of phosphorus overlap in the Israeli and Egyptian samples, Table 1). In addition, the grinding curve slope of the Herodium bathtub is exceptional. Its high value is more similar to the Egyptian values, in contrast to those from the Israeli known samples, and in contrast to most of the other results that show a similarity to samples from Israel. The grinding curve slope is dominated by relative changes in IR absorption due to different particle sizes—the product of a manual process. However, the location (offset) of a grinding curve is determined by the degree of local crystalline order, an absolute value^37^. Therefore, the offset is a more reliable indicator in determining provenance/character of the calcite. Note that the grinding curve of the Herodium bathtub was determined by seven IR peak intensities that are quite close to each other, whereas the eighth point on the graph is much farther away, presenting much higher v4 and v2 values (this exceptional result, which increases the slope of the graph, may be due to poor manual grinding of the sample). Thus, small variations in the value of this point have a very significant effect on the slope. Indeed, omission of this point would reduce the slope of the graph to 1.97, a value that is unequivocally within the range of the slopes of the samples from Israel.

## Conclusions

In this study, we developed an approach for the identification of the source of calcite-alabaster, by the combination of four analytic methods that include ten criteria. It is important to note that each method provided a clear distinction between Egyptian and Israeli material, though the provenance determination of the calcite-alabaster is strengthened by the combination of results from all these methods together.

The present study identified the origin of two magnificent royal bathtubs of Herod: they were carved from material mined in Israel. These results attest to the fact that the calcite-alabaster industry in Judea in the second half of the first century BC was sufficiently developed and of high enough quality to serve the luxurious standards of Herod—one of the finest builders among the kings of that period.

## Materials and methods

### Materials

The ancient Egyptian samples, collected by the Austrian archaeological expedition to Giza in the nineteenth century, were courtesy of the Kunsthistorisches Museum in Vienna. A modern Egyptian artifact (made of geological-sourced calcite-alabaster) was bought in a market in Cairo, Egypt in 2013.

The calcite-alabaster from Israel included raw material from the Te’omim cave quarry, chips (mining debitage) found in the cave near the quarry, and chips and a stone block (raw material) from Umm el- ‘Umdan. Additional samples were collected from a speleothem in Natuf cave. The samples from the quarry were sawn from the wall by a Makita grinder saw. All samples were crushed to powder by an agate mortar and pestle (for details of all samples and analyses, see Supplementary Table S1 online).

### Methods

The methods used in this research include: inductively coupled plasma-atomic emission spectroscopy (ICP-AES) analysis, routine infra-red (IR) spectroscopy, ^1^H- and ^31^P- solid state NMR (ssNMR) experiments and C and O stable isotope ratio analysis.

#### ICP analysis

##### Instrumentation

The amount of the following elements: copper (Cu), iron (Fe), magnesium (Mg), strontium (Sr), zinc (Zn), phosphorus (P), titanium (Ti), sulfur (S), manganese (Mn) and aluminum (Al), in each of the tested samples was characterized by elemental analysis using an inductively coupled plasma-atomic emission spectroscopy (ICP-AES) spectrometer (Ultima 2, Jobin Yvon Horiba). Classic calibration method with standard solutions was used.

##### Sample preparation

The solid samples (1gr) were dissolved in a strong acid-HNO_3_ (2 mL) at room temperature upon stirring until no stone particles could be seen in the solution. The samples were kept in a chemical hood for a week (in order to ensure complete dissolution) and then were diluted tenfold with DDW (double deionized water, 18 mL). The resulting solutions were analyzed by ICP.

##### Calibration method

ICP multi-element solution of 23 elements (Merck IV) and single elemental ICP standards with a concentration of 1000 mg/L (Cu, Fe, Mg, Sr, Zn, P, Ti, S, Mn and Al), obtained from Merck (Darmstadt, Germany), were used to prepare the working standard calibration solutions.

#### FTIR analysis

Samples were prepared from hand-ground (agate mortar and pestle, 10 s to 3 min) calcite that was diluted (~ 1:20 mass ratio) with KBr (Sigma-Aldrich, FTIR grade), then uniaxially pressed (2 tons, sufficient to allow the KBr to flow and recrystallize as an IR transparent matrix) to yield a disk that is suitable for IR measurements in a transmission geometry (Nicolet 380, 4 cm^−1^ resolution). Samples of well-defined origin and vessels were prepared as written above, and passed the grinding and measuring process 6–10 times. These measurements yielded varying v_4_ and v_2_ values, normalized by v_3_. These values were set in a graph of v_2_ vs. v_4_, to receive a set of curves with different slopes- “grinding curves”.

#### NMR analysis

^1^H, and ^31^P MAS NMR measurements were performed on a Bruker 11.7 and 4.7 T Avance III, 500 MHz, narrow-bore spectrometer, equipped with a 3.2 and 4 mm (respectively) VTN CPMAS double-resonance probe at a spinning rate of 8 kHz. ^31^P direct polarization spectra were collected with 146–11,244 scans and recycle delays of 8 s. ^1^H direct polarization spectra were collected with 160 scans and recycle delays of 5 s. Deconvolutions of ^1^H lines were carried out using the DMFIT program developed by Massiot et al.^38^.

#### Isotope analysis

δ^18^O_c_ and δ^13^C analyses were made on 0.35–0.5 mg samples, obtained by drilling at intervals of ~ 0.5–1.0 mm and put in glass vials. Dry phosphoric acid (100%) was added to the vials, which are placed in a horizontal position to avoid reaction with the carbonate. The samples were flushed with pure helium gas for 10 min in order to remove all atmospheric CO_2_, and only then the vials were turned to vertical position to allow the phosphoric acid to react with the powder of the drilled samples, forming CO_2_. The CO_2_ was measured using a Delta Plus mass spectrometer with a Gas Bench automatic sampler in order to measure δ^18^O_c_ and δ^13^C. All δ^18^O_c_ and δ^13^C values were calibrated against the international standard NBS-19, and were reported in permil (‰), relative to the VPDB standard.

## Supplementary Information

Supplementary Information.

## Data Availability

All data generated or analyzed during this study are included in this published article and its supplementary information files.
