# Publisher Correction: Sourcing Herod the Great’s calcite-alabaster bathtubs by a multi-analytic approach

**DOI:** 10.1038/s41598-022-13308-9

**Published:** 2022-06-14

**Authors:** Ayala Amir, Amos Frumkin, Boaz Zissu, Aren M. Maeir, Gil Goobes, Amnon Albeck

**Affiliations:** 1 Martin (Szusz) Department of Land of Israel Studies and Archaeology, Bar-Ilan University, 5290002 Ramat Gan, Israel; 2 Institute of Earth Sciences, The Hebrew University of Jerusalem, 91904 Jerusalem, Israel; 3 Department of Chemistry, Bar-Ilan University, 5290002 Ramat Gan, Israel; 4 Present Address: The Sonia and Marco Nadler Institute of Archaeology, Tel Aviv University, 69978 Tel Aviv-Yafo, Israel

Correction to: *Scientific Reports* 10.1038/s41598-022-11651-5, published online 07 May 2022

The original version of this Article contained an error in Figure 4 where the resolution of the Figure was low.

In addition, Table 3 contained several errors for Israeli samples (Average) and Egyptian samples (Average). The standard deviation values for “IR, slope” and the range of negative values for “Isotope ratios” were inadvertently switched.

The original Figure 4 and Table 3 and accompanying legends appear below.

**Figure 4:**
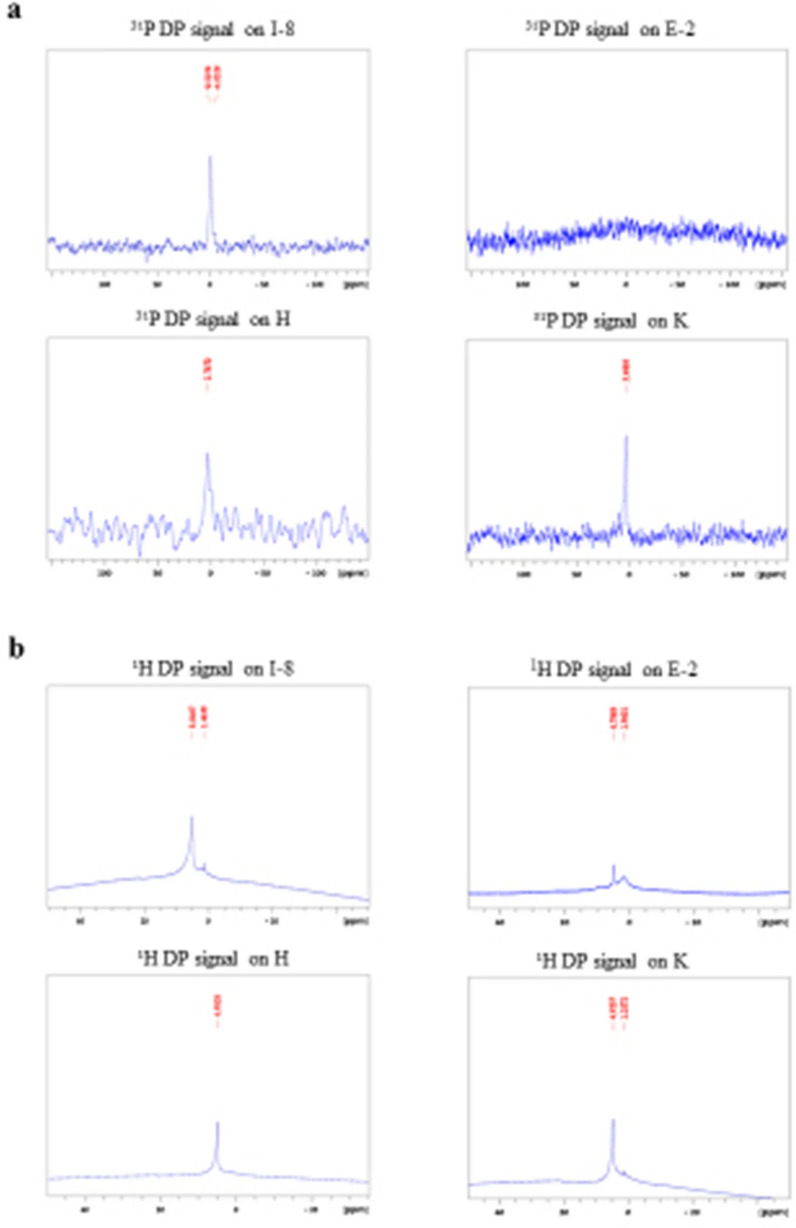
Solid state NMR results: I-8 representative sample from Te’omim cave, Israel; E-2 representative sample from Egypt; H from Herodium bathtub; K from Kypros bathtub. (**a**) ^31^P NMR data. (**b**) ^1^H NMR data.

**Table 3:**
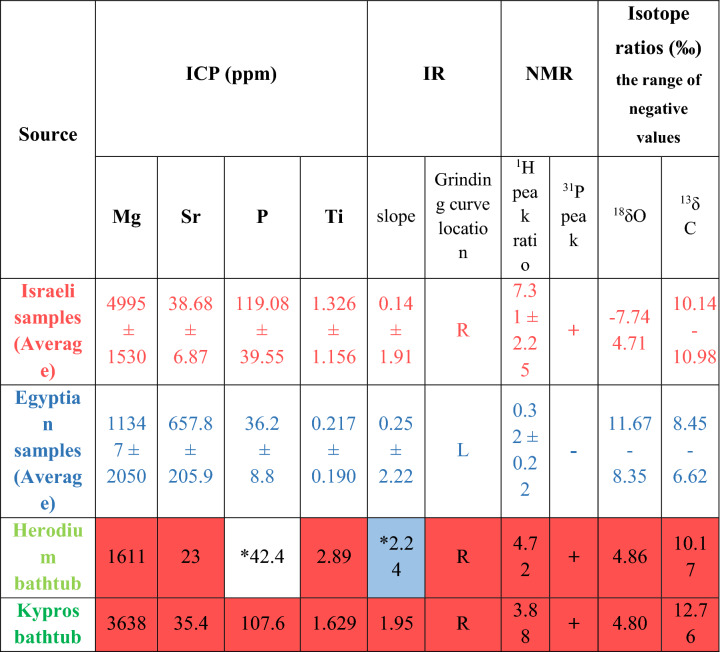
Israeli samples in red, Egyptian samples in blue. The color coding of the cells in the table showing bathtub samples indicates their similarity to the corresponding values of the "known" Israeli and Egyptian samples. *see text.

The original Article has been corrected.

