# Supplementary material for: Sourcing Herod the Great's calcite-alabaster bathtubs by a multi-analytic approach
Source: Sci Rep. 2022 May 7;12:7524. doi: 10.1038/s41598-022-11651-5 (PMC9079073; doi:10.1038/s41598-022-11651-5)

Supplementary Information for

Sourcing Herod the Great's calcite-alabaster bathtubs by a multi-analytic approach

Ayala Amir, Amos Frumkin, Boaz Zissu, Aren M. Maeir, Gil Goobes, and Amnon Albeck

Correspondence to: ayalaalbeck@gmail.com

This PDF file includes:

Table S1
Fig. S1

Table S1.
Summary of the analytical tools applied for the various Israeli and Egyptian samples.[a]

source		sample #	ICP	FTIR	NMR	Isotopes
Israel	*Teomim cave chip*	*I-1*	√	√	√	
	Teomim cave quarry	*I-2*	√			
		I-3	√			
		I-4	√	√	√	
		I-5	√	√	√	√
		I-6	√	√	√	
		I-7	√			
		I-8	√		√	
	Umm el-'Umdan chip	*I-9*	√	√	√	
		I-10	√	√		√
	Umm el-'Umdan block	*I-11*	√	√		√
	Natuf cave	*I-12*	√			√
Egypt	Giza	E-1	√			
		E-2	√	√	√	√
		E-3	√			
		E-4	√	√	√	
		E-5	√	√		√
		E-6		√	√	
		E-7				√
	Modern raw material	E-8	√	√	√	√
		E-9		√	√	

[a] Israeli samples in red, Egyptian samples in blue.

Fig. S1.

ssNMR spectra of Israeli and Egyptian samples analyzed in this research.

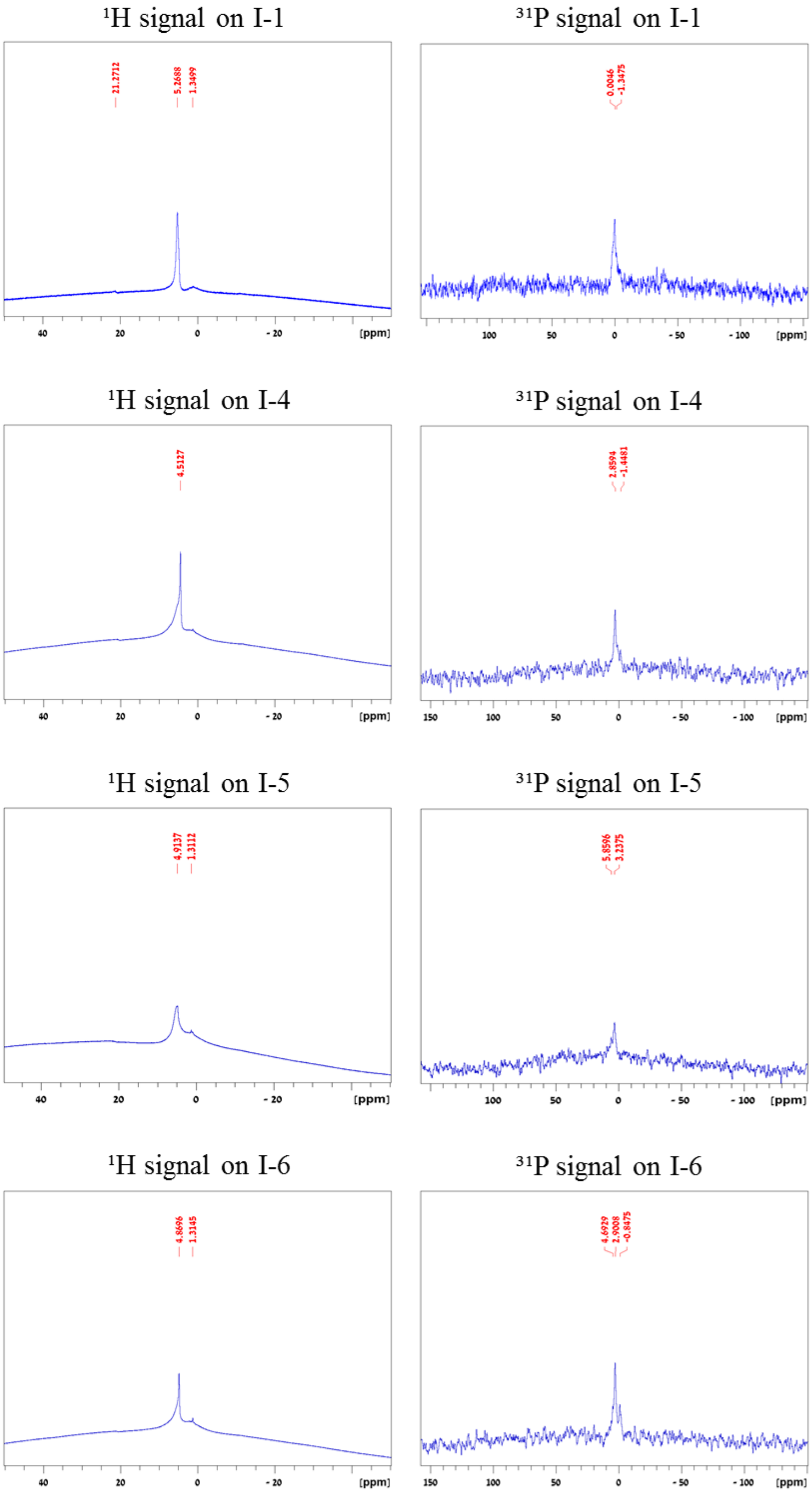

^{1}H signal on I-8

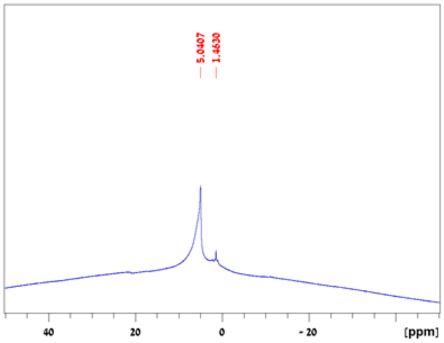

^{31}P signal on I-8

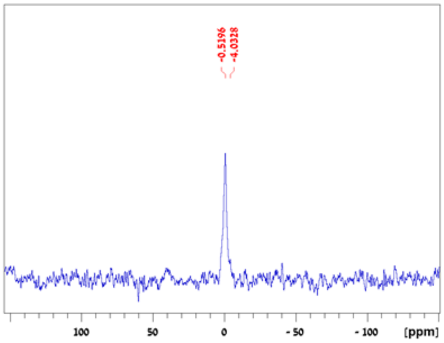

^{1}H signal on I-9

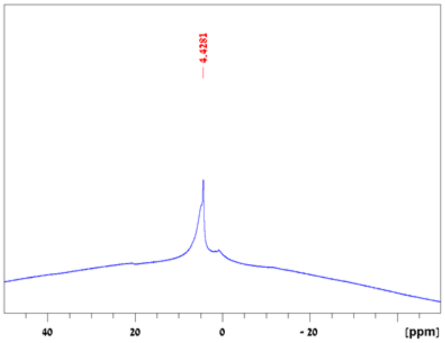

^{31}P DP signal on I-9

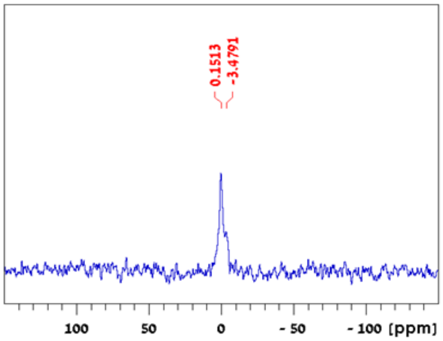

^{1}H signal on E-2

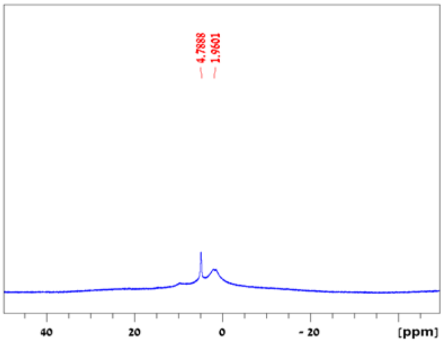

^{31}P signal on E-2

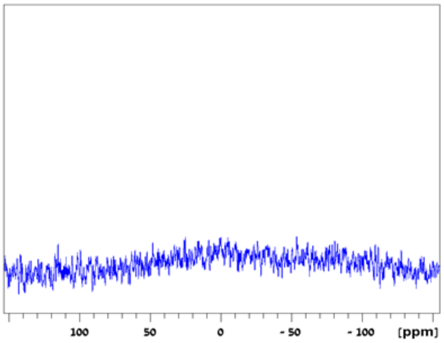

^{1}H signal on E-4

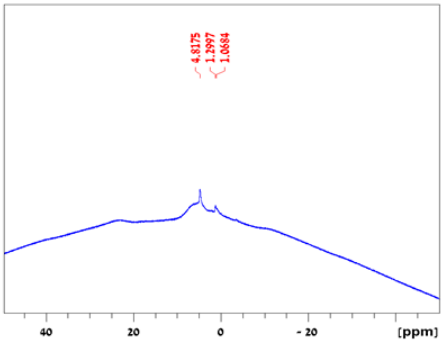

^{31}P signal on E-4

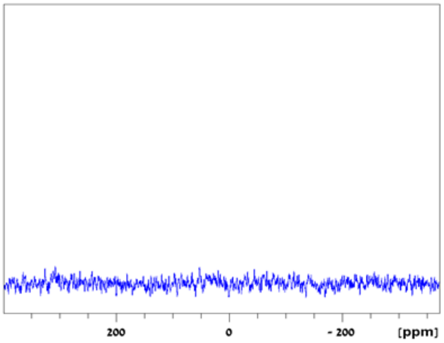

^1H signal on E-6

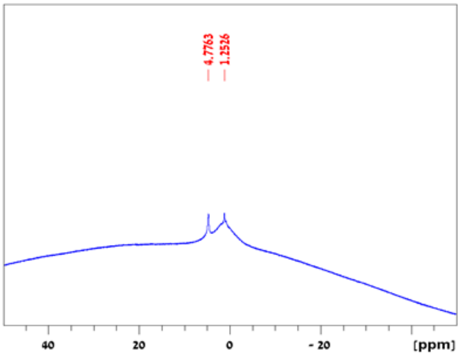

^{31}P signal on E-6

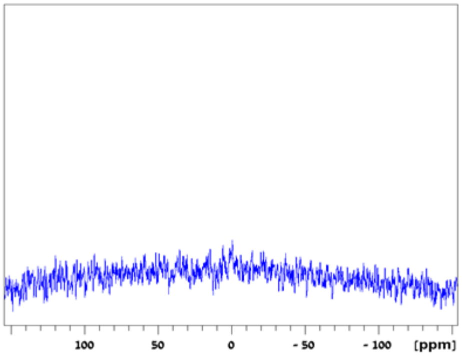

^1H signal on E-8

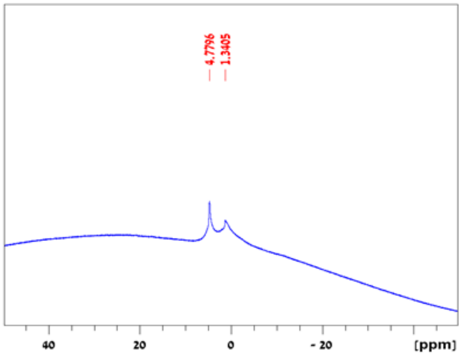

^{31}P signal on E-8

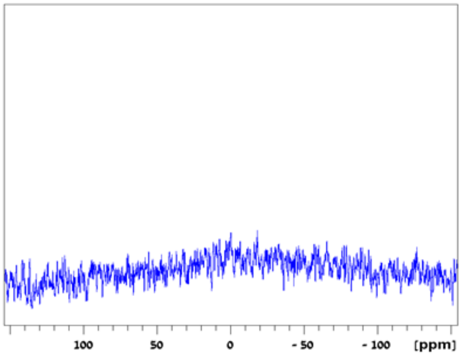

^1H signal on E-9

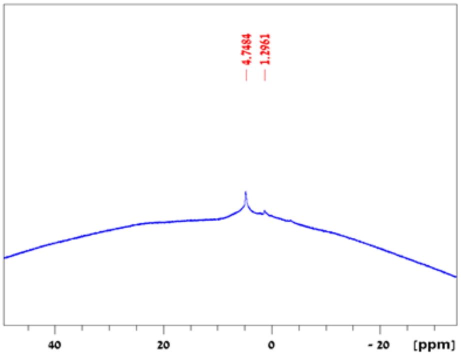

^{31}P signal on E-9

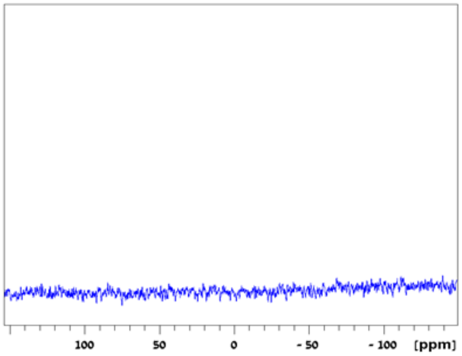

Supplement: Supplementary file 1 — Supplementary Information. [file 41598_2022_11651_MOESM1_ESM.pdf]
